# The study on designed gamified mobile learning model to assess students’ learning outcome of accounting education

**DOI:** 10.1016/j.heliyon.2023.e13409

**Published:** 2023-02-10

**Authors:** Meng-Chun Kao, Yu-Hsi Yuan, Yu-Xian Wang

**Affiliations:** a Department of Business Administration, Yuanpei University of Medical Technology, No, 306, Yuanpei Street, Hsinchu, 30015, Taiwan, ROC; b Department of Labor and Human Resources, Chinese Culture University, No. 55, Hwa-Kang Rd., Yang-Ming-Shan, Taipei, 11114, Taiwan, ROC

**Keywords:** Accounting education, Game-based learning, Mobile learning, IS success Model, BOPPPS

## Abstract

This study constructs an innovative course for accounting teaching based on a student-centered strategy. The curriculum is designed through the effective teaching module (BOPPPS) to assist students to understand accounting knowledge. A game-based mobile learning environment is created by developing an accounting mobile game and combining it with a mobile learning system (TronClass). The private technology university students had been selected by purposive sampling. A total of 81 accounting majored students, among them, 41 students are in the experimental group, and the rest were in the control group. The quasi-experimental design was to be applied in curriculum development. Meanwhile, quantitative data were collected by questionnaires and the qualitative data were collected through interviews. The result shows that game-based mobile learning can be beneficial to teaching effectiveness. And the regression model supports that information quality and service quality have positive predictive power on use intention. In addition, use intention and user satisfaction impacted learning engagement positively. Further, user satisfaction has a mediating effect. Finally, some suggestions are put forward to provide references for accounting educators and researchers.

## 1 Introduction

In the era of advanced technology, mobile networks are popular, and the functions of mobile devices are changing with each passing day to improve the convenience of mobile phones. In addition to the basic functions such as calling, taking pictures and videos, and other built-in functions of mobile phones. Current smartphones have many other functions, such as instant messaging, social media, online browsing information, learning, watching videos, listening to music, playing mobile games, etc. [1]. With increasingly new mobile phone functions and applications being developed, it has become an essential tool in daily life. The Taiwan Communication Survey Database [2] analyzed Taiwanese use of mobile phones. The report pointed out that 88.2% of people used mobile phones daily. In Taiwan, everyone had his or her own mobile phone and the average usage time was nearly 3 h per day. On an increasing trend, people aged 18–29 spent more than 5 h using their mobile phones. Especially the average time spent on the mobile phone is as high as 5 h and 13 min per day for respondents aged 18–19. In other words, more than 1/5 of the day was occupied by mobile phones, showing that the student group was heavy mobile phone users. If the mobile phone could be applied to the teaching field reasonably, it would become a powerful mobile learning tool. Indeed, educators could combine online learning platforms and wireless communication devices in online teaching and learning [[3], [4], [5]]. Mobile learning was born, and the teaching model has changed dramatically. This new type of learning provides learners with the possibility of “wireless” and ubiquitous learning opportunities [[5], [6], [7]]. Anyone who is willing to learn can study at anytime and anywhere to enrich personal knowledge efficiently [8].

Moreover, thanks to the popularity of mobile devices, mobile games have become the largest market category in the global game industry. More than half of the total revenue of the game industry comes from mobile games [9,10]. Kantar Insights Taiwan [11] conducted a survey on netizens aged 16–65 in Taiwan in 2019. It showed up to 70% of the respondents were mobile game players and 44% were even heavy gamers who played every day. More than half of the younger group had a higher penetration rate of mobile games. In the teaching scene, it is often seen that students are addicted to playing games in the mobile world. They cannot drop their mobile phones to concentrate on their studies even in class. If students are not prohibited from using mobile phones, it might be tried to integrate them into the learning environment. Moreover, Seow & Wong [12] incorporated the mobile game into the accounting teaching scene and received positive feedback. Therefore, the researchers should think about how to make mobile phones helps rather than a hindrance to teaching.

Game-based learning has flourished in recent years. Most research results directly support that game-based learning can improve learning motivation and effectiveness [[13], [14], [15], [16], [17], [18], [19]]. However, the application of research in accounting education is still very lacking [19]. The game-based learning has a positive effect on accounting learning that supported by previous studies (e.g. Refs. [12,[14], [15], [16],^18^,^20^]). Yet, it is not fully applied in the higher education for accounting curricula. The reason is that there are few learning games suitable for accounting disciplines at present. Further, it is difficult to collect relevant empirical data, resulting in few applications of game-based learning in accounting education research. [19,21,22], thus the difficulty of its promotion is increased.

This study applies game-based mobile learning to the mobile research of accounting courses, and it constructs innovative curriculum plans. The learners' learning achievement was the goal of this study, and professional accounting knowledge was the observation variable in this study. The BOPPPS (bridge in, outcomes, pre-assessment, participatory learning, post-assessment, and summary) teaching model [23,24] has been employed in this study. It combines game-based learning and mobile learning to create a game-based mobile learning environment. In the implementation process, the researchers pay attention to curriculum design, teaching methods, learning assessment, curriculum reflection, and review loop mechanism. Further, the researchers carry out continuous corrections to solve the problems in the teaching scene. It may reach the goal of excellent teaching and training targets. The research object of this study is the students who took accounting courses in the division of continuing education of selected private technology universities in Taiwan. Through the quasi-experimental design, the researchers will understand the influence of game-based mobile learning on learning outcomes. It explores the success factors affecting game-based mobile learning with the information system success model. In addition, the purposes of this study are.

(1) Understand the current situation of the barrier of accounting learning,
(2) The reasonable structure of the accounting learning game,
(3) The learning outcome of the game-based leaning course,
(4) Developing a useful game-based accounting learning course.

## 2 Literature review

### 2.1 Game-based mobile learning

Game-based learning allows students to learn in the context of games. Participating students must abide by the rules of the game, and the competitive environment will enhance the willingness to actively participate in learning. Focus on finding problems and find effective solutions to problems in the process to acquire knowledge in cognitive learning [25,26]. When it comes to digital game-based learning, it is necessary to design an educational game that can combine teaching content and game characteristics. Further, this game can immerse people in facing challenges and repeatedly trigger a positive cycle of judgment, execution, and system feedback. In the end, the learning goals are achieved by investing in such games [27,28]. It is mentioned that the use of game situations can arouse students' curiosity. At the same time, it increased students' intrinsic motivation and interest in learning and further improve students' learning effect [29,30]. Nitkin [20] proposed strategies for introducing accounting cycles and encouraging cooperative learning through games.

After the learners go through the teaching design of the “Life Chemistry Online Game Learning System”, significant improvement in learning achievement. Mobile learning is a brand-new concept that is different from traditional learning, and it allow to interact with other devices through mobile devices and wireless networks [31]. Because mobile devices are easy to carry and portable and integrated with wireless networks. Learners can learn at the right time and place, and enjoy the convenience, immediacy, and suitability of mobile technology [32,33]. This indicates that mobile devices are often used in education. In addition, mobile learning combined with big data and mobile technology has become more useful in education applications [34].

It indicated that mobile learning could help learners overcome learning obstacles anytime and anywhere in the learning process. In addition to the advantages of traditional distance teaching, it can also effectively solve problems. Mobile learning has been widely used and has become a prominent study in educational research at home and abroad [35]. A summary of previous research points out that mobile learning is based on mobile products, learning with mobile network support, and other conditions [15,16,36]. Learning is not limited by time and space and can be continued anytime, anywhere. Therefore, mobile learning meets the urgency of learning needs and makes learning content readily available. With the convenience of the internet, learners can actively search for information on the internet from everywhere, obtain useful knowledge, become more mobile, and achieve the characteristics of “ubiquitous”, which increases the convenience of learning [32,36].

One of the challenges faced by current mobile learning is the lack of mobile learning teaching materials. If only the original written teaching materials or digital teaching materials are transferred to the mobile vehicle for presentation. The results were only to take the original teaching materials with them. It is only attractive to students, their motivation and interest in learning are like those in traditional classrooms. They cannot give full play to the advantages of mobile learning [5,37]. Mobile learning allows learners to have great flexibility and freedom. For those who are not enthusiastic about learning, it may become a good opportunity for laziness and failure to achieve their learning goals. Even if there is a passion for learning, there is still the ability to learn methods and time management. Therefore, how stimulating the enthusiasm for learning and enabling students to learn independently and obtain the benefits of digital learning is a critical issue that mobile learning needs to face.

If teachers in the future can improve their professional knowledge and innovative teaching ability, they should integrate new-age information technology into the existing curriculum structure and teaching connotation to enrich their teaching and create innovative teaching. That conforms to the digital learning model and cultivate talents who can meet the trend of the new generation [10,38]. To overcome the dilemma of mobile learning, many scholars have proposed a new learning model combining “game-based learning” with “mobile learning” [39]. When designing a set of game-based mobile learning, the most crucial step is to appropriately present the knowledge or training unit to be constructed and imported into the context of the mobile game [40]. Then it does not destroy the fun of the game but can use the game immersion method to achieve the purpose of learning and training skillfully [29,41].

Indeed, put forward and analyze the concept of mobile learning combined with simulation games, combined with the learning mode of operating virtual mobile in a real environment, which can enable learners to expand their learning experience and improve their learning motivation [4,17]. Use games on mobile devices to construct a cooperative learning environment related to local urban history. So that learners can be immersed in the learning process by playing distinct roles and effectively improve learning motivation [13]. The developed English learning system on hand-held devices provides students with English teaching materials related to their surrounding environment on campus. The research results confirm that the learning environment is better than a non-mobile game learning environment for learning motivation and learning effect [42].

McDonald's in Japan introduced Nintendo game consoles to replace the method of training hourly employees with traditional manuals. It is allowing employees to master the knowledge required for work quickly through interactive games to effectively reduce training costs [43]. A summary of previous research points out that game-based learning can transform learners' motivation to participate in game activities into motivation to participate in learning activities. Thereby, improving learners' learning effectiveness that the impact of game-based learning on learners lies in triggering intrinsic motivation for learning. In this interactive environment, learners conduct independent or group operational activities. Students learn happily can enhance their sense of achievement and improve their learning effectiveness [6,31]. Seow & Wong [12] introduced the first mobile game for accounting learning, allowing students to learn accounting by answering questions and improving their learning motivation. Most of the user feedback is satisfied with this mobile game.

Nowadays, most textbooks on mobile learning are based on commercial considerations and few are produced for specific learning subjects. It results in the general shortage of learning software that really uses the characteristics of mobile vehicles [3,8]. There is a lack of teaching software that combines games with mobile learning. In this study, accounting knowledge is put into the game in a complete and contextual form, and more story-like situations and challenging levels are integrated into the practice questions for learners to break through the levels. After learning the necessary knowledge of this unit, the most important thing after arousing learning motivation is to maintain the learning motivation. By strengthen the interest and diversity of learning and improve the learning effect.

### 2.2 Delone and McLean IS success model

**Fig. 1:**
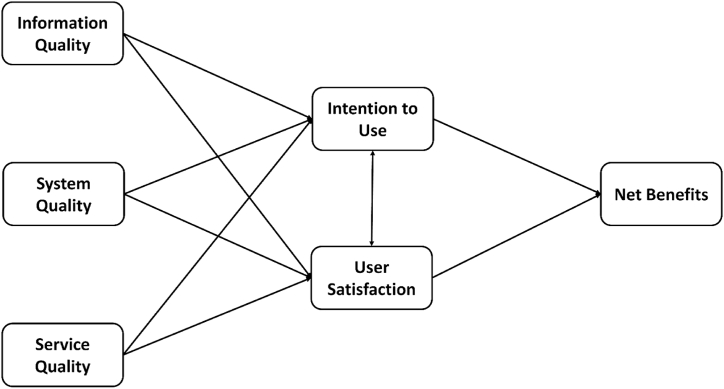
The information system success model.

Source: DeLone & McLean (2003).

DeLone & McLean [44] proposed an information system success model to evaluate the impact of an organization's use of information systems, including six dimensions: System quality, information quality, system usage, user satisfaction, personal impact, and organizational impact which revised in 2003. In the original model, the aspect of service quality is incorporated into the original model, and the two aspects of personal impact and organizational impact are combined to call the net benefit. Temporal and cause-and-effect models are proposed for the interrelationships measured between the various indicators, and the identification of stakeholder groups at stake in the measurement process begins. “Information quality”, “system quality” and “service quality” individually or simultaneously affect “intention to use” and “user satisfaction”. In addition, “use intention” and “user satisfaction” will affect each other, and the two dimensions of “use intention” and “user satisfaction” will affect the “net benefit”. This is also the first time measuring the success of an information system introduced the concept of a certain order [45,46]. Discuss the impact on the intention to use the system and user satisfaction, and its mode structure is shown in Fig. 1.

The various aspects of the information system success model are introduced and explained as follows.

• *Information quality*: A measure of the quality of information produced by an information system which including the correctness, reliability, completeness, relevance, timeliness, clarity, understandability, usefulness, and up-to-datedness of the output information.
• *System quality*: Refers to the evaluation of the system quality of the information system itself. To measure whether the system has its due characteristics, including ease of use, reliability, availability, adaptability, and response time of the system.
• *Service quality*: Refers to the overall service provided by the system service provider, or the quality of the service provider which including the responsiveness, guarantee, immediacy, and consideration of service provision.
• Intention to use: It is used to measure whether the user is willing to use the information system, and the use of the information system. Such as system use, system operation, information acquisition, and execution which including ease of use, degree of guidance, use time, use frequency, use voluntariness and conversion processing times, etc.
• *User satisfaction*: Refers to whether users are satisfied with the information system after using the information system. The proposed measurement items are decision satisfaction, hardware satisfaction, software satisfaction, overall satisfaction, information satisfaction, and interface satisfaction. degree, etc.
• *Net benefit*: Refers to the benefit of the information system to the user itself and the performance of the organization which including improving the work efficiency of personnel, service quality, communication effect, overall image, effectively saving manpower, and reducing operating costs, etc.

In addition, the definition of learning engagement is the degree to which students actively participate in and engage in learning activities of the learning process [47]. Webster & Ahuja [48] emphasized that user input is an important indicator of the future use intention and performance of the system. Lalmas, O'Brien, & Yom-Tov [49] considered that user engagement is an important concept in designing online applications. Regardless of whether desktop, tablet, or mobile systems are used successfully and the motivations for successful applications include usage and engagement. Emphasizing active engagement enables students to actively adjust and enrich their learning process in what they are learning, learning conditions, and environments.

This study attempts to explore the key success factors of students' use of game-based mobile learning. When students use games to learn, improving the information quality, system quality and service quality in the process of students using the game-based mobile learning system can effectively enhance students' willingness to continue using the system in the future [50]. Therefore, this study applied the information system success model incorporating the learning engagement dimension to evaluate the factors that affect students' learning engagement [51].

### 2.3 Learning game design

The teaching model “BOPPPS” is widely used in curriculum design for game-based learning. Each English letter is an abbreviation of teaching activity, namely: B is Bridge-in, O is Objective or Outcome, first P is Pre-assessment, second P is Participatory Learning, third P is post-assessment, and S is Summary. Follow the objective laws of teaching activities to obtain as much teaching effect and benefit as possible with as little time, material resources and energy as possible.

According to the learner's conditions, students are instructed in accordance with their aptitude. Learning activities are designed according to the content theme, and various classroom interactive modules are used to enhance the learning effect. Lesson plans and teaching drills are developed in accordance with learners' ability, willingness, and teachers' expectations to obtain effective, efficient, and effective teaching results [52]. This research follows the effective teaching module model, the accounting knowledge in the course is closely combined with the game and the accounting mobile game is developed. The content of the course is the secret of game clearance, to help students defeat evil, and put the course materials on the mobile learning platform (TronClass). So that Students can learn independently according to their own situation.

The teaching topics include the following five subjects: Basic concepts of accounting, accounting entries, period-end adjustments, financial report preparation, period-end closing procedures, and accounting for the trading industry. In the basic concepts of accounting, basic accounting theory, terminology, and accounting items are taught. Thus, students can learn in the game through the accounting mobile game - collecting elements and tasks for the elderly.

**Fig. 2:**
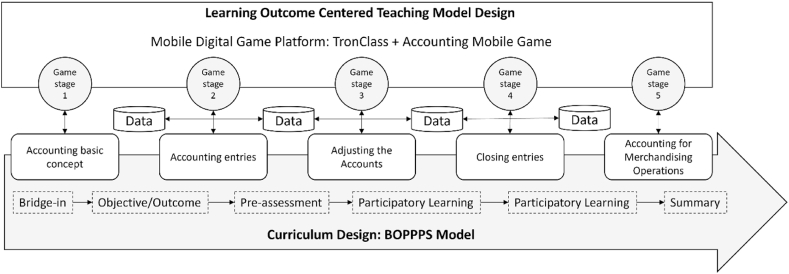
The concept of this study.

The second topic is accounting entries. Using the accounting mobile game “Synthetic Elf”. Students can familiarize themselves with accounting rules and accounting items, and then be able to write reasonable entries and explain transaction matters. The third theme was an adjustment that through the accounting mobile game - Helping the “Tiaogi Clan”. Hence, students become familiar with the adjustment points and methods and then be able to write adjustment entries. The accounting mobile game - Fight against Evil, let students learn about checkout assignments (the fourth theme) and accounting for the trading industry (the fifth theme) to familiarize themselves with accounting knowledge. In addition, through the holding of golden note selection and game competitions. Therefore, students can increase their learning input and deepen their learning effectiveness, and conduct assessments, feedback sheets and questionnaires in line with the course content. Further, the collected quantitative and qualitative data and conduct course reviews and reflections was conducted in the course. The problems found in the teaching site are adjusted and corrected, and the curriculum improvement mobile is actively carried out. Specifically, the concept of this study is shown in Fig. 2.

In addition, the exploration of research gap of this study is focused on the learning outcome and its effectiveness toward designed accounting learning game and the game-based accounting education. Further, the novelty of this study is to discover the performance of game-based accounting learning that still lack on current accounting education by designed accounting game as mentioned before.

### 2.4 The hypotheses development

Based on the literature argument, the information quality will affect the users’ intention to use the system, especially in the mobile learning environment [53]. Thus, the hypothesis was setup accordantly.

H1 The information quality will impact the users' intention to use positively.

Further, the system quality could influence the users’ intention to use the e-learning system obviously [54]. Thus, the hypothesis was established.

H2 The system quality will impact the users' intention to use positively.

Moreover, the service quality of the system will affect the users’ intention to use it as well. E.g., Mugion, Toni, Raharjo, Di Pietro, & Sebathu [55] survey the citizens of Rome, Italy. The result demonstrated that users will be affected by service quality significantly. Thus, the hypothesis was established.

H3 The service quality will impact the users' intention to use positively.

Indeed, Wan, Xie, & Shu [56] found that user satisfaction with MOOCs will be influenced by students’ intention to use the system dramatically. Thus, the hypothesis was established.

H4 The users' intention to use will influence user satisfaction toward the system positively.

Bitrián, Buil, & Catalán [57] argued that gamification enhances users' engagement with their mobile apps, and the users’ satisfaction with the used system will impact their engagement directly. It was supported in their study. Thus, the hypothesis was established.

H5 The user satisfaction will influence users' learning engagement positively.

H6 The users' intention to use will influence users' learning engagement positively.

## 3 Research methodology

### 3.1 Research design and framework

This research explores whether the use of accounting mobile games and mobile teaching platforms combined with game-based learning and mobile learning in accounting teaching. It gained a positive impact on college students learning engagement and learning effectiveness. This study used a quasi-experimental design method of one-group pretest-posttest design shown in Table 1.

**Table 1:** Quasi-experimental design table.

Group	Pre-measurement	Experimental intervention	Post-measurement
experiment group (n = 41)	O1	X1	O2
control group (n = 40)			O3

Note: O1 is the knowledge test before the experiment; X1 is a five-week gamified learning implementation; O2 is the test and questionnaire after the experiment; O3 is the post-test of the control group.

The research objects were divided into an experimental group and a control group. The experimental group used TronClass combined with the game learning platform to integrate the accounting teaching method. The control group only maintained the original general traditional teaching method. The experimental period was five weeks in total. To verify the effectiveness of game-based mobile learning in accounting courses, the independent variable was game-based mobile learning. The dependent variables were the test scores of learning effectiveness. Which were the factors affecting the use of the accounting mobile game and learning engagement. And the control variable was that all learners have the same number of teaching hours, learning objectives, and learning materials, etc. In the collection of learning effectiveness data, before the course, the experimental group was given a subject test as a pre-test. After the curriculum incorporated game-based mobile learning, the subject test was given as a post-test. Through the analysis and comparison of pre-test and post-test results, the impact of game-based mobile learning on students' learning effectiveness was explored. In addition, the control group was also given the subject test after the course to compare the impact of different teaching methods on students' learning effectiveness.

After the experimental teaching, students were asked to fill in a questionnaire to understand the factors that affect students' game-based learning motivations and perspectives. The questionnaire consisted of six dimensions of information quality that were system quality, service quality, intention to use, user satisfaction, and learning engagement. The original questionnaire was modified from DeLone & McLean [50] in terms of information quality, system quality, service quality, intention to use, and user satisfaction. The reliability coefficient of the original questionnaire was 0.92.

**Fig. 3:**
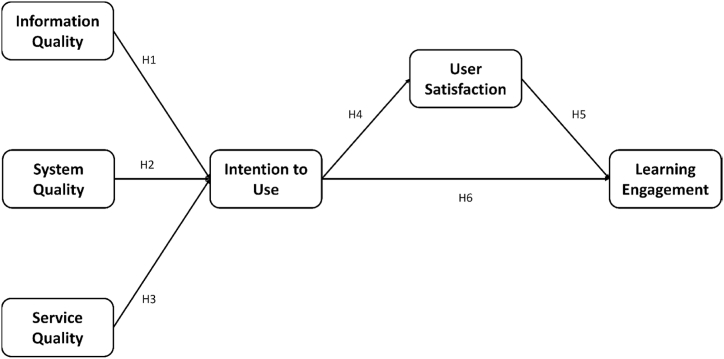
Hypothesis model.

Learning engagement was defined as the time and effort devoted by students to educationally purposeful activities, which could be further divided into behavior, emotion, and cognition. The behavioral engagement was the explicit manifestation, and emotion and cognition were the inner psychological manifestation [[58], [59], [60], [61]]. The questionnaire questions in measuring learning engagement dimensions were modified by Blasco-Arcas et al. [59]. The Likert five-scale scale was used, including 4 items on information quality, 3 items on system quality, 3 items on service quality, and 3 items on intention to use. The reliability coefficient of the original questionnaire was 0.94. The Cronbach's α value of the overall questionnaire in this study was 0.985, and the Cronbach's α value of each dimension was above 0.8 which indicated the questionnaire had good reliability. In addition, qualitative information was collected by the study feedback form filled out through the students. Fig. 3 describes the independent and dependent variables.

#### 3.1.1 Game unit design and subject correspondence

**Fig. 4:**
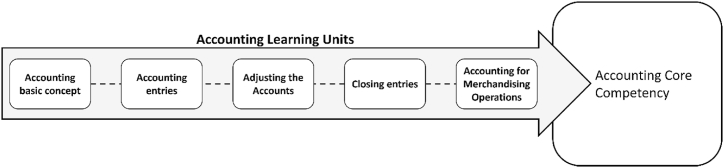
The teaching units of accounting learning units.

In terms of content, this study takes accounting as the research scope, and the curriculum is planned as a series, from the basic concepts of accounting, the complete accounting cycle, business accounting, etc. And it is planned into five major topics as shown in Fig. 4.

This study developed the accounting mobile game combined with the theme of the course. The researchers constituted stories, scenes, and characters through different accounting themes to increase game tasks and improve the richness of the game. Meanwhile, the design of gamification mechanisms such as peer competition, feedback, and rewards could provide learning incentives. These features make students more expectations and fresh for the game, so as to increase the stickiness of the game. Gamification could enhance learning motivation and encourage learning engagement [[62], [63], [64]].

The detailed accounting curriculum combined game-based mechanism was, first, the teacher explains and interprets the accounting unit. Then students download the app from the internet and register as players in the designed accounting game. Thus, students can access the specified stage corresponding to the teaching unit and start the game stage. This teaching model can help students be familiar with and become proficient in accounting knowledge. The combination of course themes and game design were shown in Table 2.

**Table 2:** The screen shot of the designed accounting game.

Designed game units	Game units	Accounting learning units
	Gathering Elements - Recognizing Accounting Items	Accounting item classification
	Cultivating elves - Law of Accounting	Accounting Entry Highlights
	Help the tangerines - adjust the focus	Adjust entry focus
	Redeem Blessing - Checkout Item Identification	Checkout method
	Combat - Familiarize yourself with accounting	Accounting knowledge such as entry, adjustment, closing and trading accounting

### 3.2 Curriculum design

The curriculum design is shown in Table 3. A total of five weeks of experimental teaching activities are implemented. The selected textbooks and the self-edited teaching materials were placed on the online digital teaching platform and matched with accounting mobile games to create a game-based mobile learning environment. It allowed students to learn in the game situation. The students could master professional accounting knowledge and skills through repeated practice at anytime and anywhere.

**Table 3:** Course topics and schedules.

Week	Class subject	Week	Class subject
W1	Course Introduction/Research Quiz Pre-Test	W10	Adjust concept and focus
W2	Basic Concepts of Accounting	W11*	Adjusting Entry Types and Implementation
W3*	Accounting item	W12	Financial Statements
W4	Accounting cycle	W13	Checkout Concepts & Highlights
W5	Lending Law and Double Entry Bookkeeping	W14*	Checkout implementation
W6*	Accounting entry implementation	W15	Business Accounting
W7	Posting Methods and Implementation	W16*	Business accounting practice
W8	Trial method and practice	W17	Review/Research Questionnaire
W9	Midterm exam	W18	Final exam

Note: * for game teaching.

The method of course implementation was based on the online digital teaching platform to build accounting knowledge chapters as the curriculum foundation. Further combined with the accounting mobile game which developed according to the teaching content. The procedure of this research was that before the course started, a pre-test was carried out to understand the students' prior knowledge and learning initial behavior. Through the game process, students must memorize and apply them before they can cope with the knowledge point challenges of the game level and complete the game tasks. Through the game, students could become proficient in accounting learning units and knowledge points, and help students construct their own accounting knowledge.

In the five-week experimental teaching, each student played the accounting mobile game for 1 h each week in the classroom to implement game-based learning. The researchers collected data on students' learning outcomes for statistical analysis and verification to understand students' learning outcomes. The control group was taught in the traditional way of lecture, and the teaching content, tests, and hours were the same as those in the experimental group.

### 3.3 Participants

The subjects of the experimental group were sophomores who were taking the accounting course in the continuing education division of the Department of Business Administration. A total of 46 students participated in the course, completed the quiz, and answered the questionnaire. Among them, males accounted for 48.8%, females accounted for 51.2%, and females were slightly more than males.

It is found that 75.6% of the students have first time to learn accounting, only 24.4% were experienced. Then only four students had accounting certificates. There were only 22% from business management groups. In addition, 80.5% of the students liked to play mobile games. As shown in Table 4, total 19.5% of the students did not like to play mobile games and 65.9% of the students usually play mobile games. Among the 40 students in the control group, 24 females were the majority (60%), 17 students have taken the accounting course, and only two students had accounting certificates.

**Table 4:** Sample data summary table.

Variable name	Project	Visits	Percentage	Cumulative percentage
Gender	Male	20	48.8	48.8
	Female	21	51.2	100.0
Have you taken an	Yes	10	24.4	24.4
accounting course	No	31	75.6	100.0
Do you have an accounting certificate Group	Yes	4	9.8	9.8
	No	37	90.2	100.0
	Business management	9	22.0	22.0
	Restaurant	13	31.7	53.7
	Foreign language	2	4.9	58.5
	Others	17	41.5	100.0
Do you like to play mobile games	Like very much	4	9.8	9.8
	Like	21	51.2	61.0
	Ordinary	8	19.5	80.5
	Do not like	8	19.5	100.0
Do you play mobile	Yes	27	65.9	65.9
games	No	14	34.1	100.0

## 4 Ethics statement

The studies involving human participants were reviewed and approved by Yuanpei University of Medical Technology, Hsinchu, Taiwan. The patients/participants provided their written informed consent to participate in this study.

## 5 3.6 author contributions

1 - Conceived and designed the experiments: Meng-Chun, Kao, and Yu-Hsi, Yuan, 2 - Performed the experiments: Meng-Chun, Kao, and Yu-Hsi, Yuan, 3 - Analyzed and interpreted the data: Meng-Chun, Kao, Yu-Hsi, Yuan, and Yu-Xian, Wang, 4 - Contributed reagents, materials, analysis tools or data: Meng-Chun, Kao, Yu-Hsi, Yuan, and Yu-Xian, Wang, 5 - Wrote the paper: Meng-Chun, Kao, Yu-Hsi, Yuan, and Yu-Xian, Wang.

## 6 Results and discussions

### 6.1 The impact of game-based mobile learning on learning outcomes

At the beginning of learning, students are given a pre-test. After introducing the game-based mobile learning method, a post-test is given at the end of the semester. After that, in each course theme, in the same way, subject tests are given before and after the class. The pre and post-test scores were analyzed and compared by paired-sample *t*-test. The results showed that the post-test scores were significantly higher than the pre-test in each item (see Table 5). It indicates that game-based mobile learning can help improve learning outcomes.

**Table 5:** The effect of game-based learning on learning outcomes Pre-test and post-test mean paired sample *t*-test coefficient summary table.

Project	Number	Average	Standard deviation	T value
Accounting project post-test	41	71.95	26.098	12.048**
Accounting Project Pre-Test	41	35.37	27.577	
Post-entry test	41	70.49	19.487	13.828**
Pre-entry test	41	34.39	20.006	
Post-adjustment	41	69.27	25.114	14.603**
Adjust the pretest	41	33.05	25.712	
Checkout posttest	41	71.22	17.349	9.815**
Pre-checkout	41	36.59	24.760	
Accounting post-test in the trading industry	41	73.90	19.220	14.535**
Pre-assessment of trading accounting	41	33.17	20.909	
Accounting full post-test	41	69.41	21.951	12.378**
Accounting full pre-test	41	29.71	19.399	

Note: **p < .01.

To further compare the discrepancy of post-test mean score between experimental group and control group, the independent sample *t*-test was used to check it (see Table 6).

**Table 6:** Summary table of independent sample *t*-test coefficients for the post-test mean of the whole unit of accounting in the experimental group and the control group.

Group	Number	Average	Standard deviation	T value
Test group	41	69.41	21.951	3.766***
Control group	40	51.00	22.049	

Note: ***p < .001.

The average post-test mean score of the experimental group is 69.41, which is significantly higher than that of the control group.

### 6.2 Information system success model explores factors influencing game-based learning

#### 6.2.1 Descriptive and correlation analysis among factors

This study added the learning engagement dimension according to the success model of the information system [50] and a proposed structural equation model was established. To deal with the potential error of bias or validity threat caused by small samples and/or abnormal distribution. Partial least squares (PLS) and bootstrapping were applied [65,66] to explore the relationship between information quality, system quality, and service quality on learning engagement, user satisfaction, and intention to use. To understand whether game-based mobile learning affects learning engagement and user satisfaction and whether it affects students' intention to use the mobile game. The descriptive statistics of each research variable and the results of the Pearson correlation coefficient analysis (see Table 7). The scores of each dimension are above 4 points, and there is a significant positive correlation between each dimension.

**Table 7:** List of narrative statistics and correlation analysis (n = 41).

Structure	1	2	3	4	5	6
**1. Information quality**	─					
**2. System quality**	.827**	─				
**3. Service quality**	.812**	.919**	─			
**4. Intention to use**	.879**	.876**	.899**	─		
**5. User satisfaction**	.807**	.850**	.899**	.843**	─	
**6. Learning engagement**	.940**	.886**	.900**	.948**	.889**	─
**Average:**	**4.409**	**4.350**	**4.415**	**4.301**	**4.457**	**4.372**
**Standard deviation:**	**0.689**	**0.687**	**0.706**	**0.748**	**0.622**	**0.698**

Note：*p < .05; **p < .01.

#### 6.2.2 The verification result of hypothesized model

In this study, confirmatory factor analysis (CFA) was used to test the measurement mode. The factor loadings of all observed variables for their individual latent variables ranged from 0.876 to 0.977. These values reach above the proposed threshold of 0.45 [67]. In terms of average variation extraction, the average variation extraction (AVE) of the six facets is all greater than 0.50. Higher AVE of the facet indicates that the facet has a higher convergent validity. It shows that the scale of this study has a considerable degree of convergent validity [67,68]. The values of compositional reliability, Cronbach's α and the rho_A are all above 0.80, indicating that the dimensions are reliable. The reliability and validity of each of the dimensions is shown in Appendix 1.

**Fig. 5:**
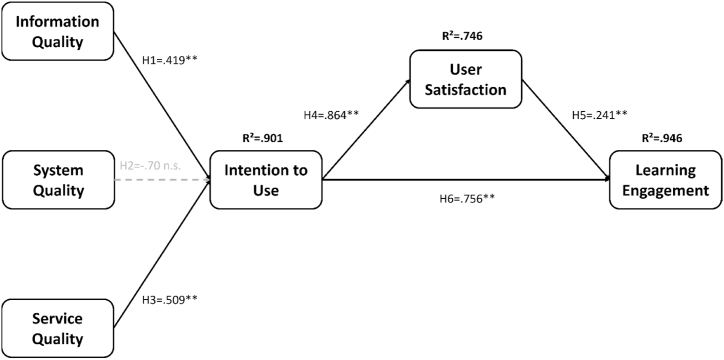
The test results of the hypothesized model with standardized path coefficients. Note: n.s. *p* > .05; *p < .05; **p < .01.

The parameter estimation results of the theoretical model are as follows. Information quality (*β* = 0.419, *p* < .01) and service quality (*β* = 0.509, *p* < .01) have a positive and significant impact on usage intention (R^2^ = 0.901). System quality (*β* = 0.070, *p* > .05) was no influence on usage intent. The intention to use (*β* = 0.864, *p* < .01) has a significant positive correlation with user satisfaction (R^2^ = 0.746). Intention to use (*β* = 0.756, *p* < .01) and user satisfaction (*β* = 0.241, *p* < .01) significantly and positively affect learning engagement (R^2^ = 0.946). The research results show that the information quality and service quality provided by this accounting game-based mobile learning system has a significant positive impact on the use intention. The higher the students' use intention is, the higher the user satisfaction. And the use intention has a significant impact on learning engagement. In addition to the direct enhancement effect, user satisfaction also has a partial mediating effect on learning engagement (Sobel Test = 2.788, *p* < .01) [69]. The relationship between the structural equation model variables is shown in Fig. 5 below.

### 6.3 Qualitative learning feedback

#### 6.3.1 Game-based mobile learning is not limited by time and space

Students feedback that he can use it anytime, anywhere, making learning more convenient:

*“Mobile games can help you learn to use without being limited by a computer*"(Interview Record Code: S-20-03)

*“You can discuss with your friends and classmates at any time, without the limitation of use time, you can start learning and use at any time when you are free”* (Interview Record Code: S-12-03)

*“I think it's not bad to use mobile games to help study”* (Interview Record Code: S-10-04)

*“Convenient, you can practice wherever you go, and the music is full of good music”* (Interview Record Code: S-05-02)

#### 6.3.2 Game-based learning stimulates interest and reduces study pressure

Students think that the course is interesting and lively, which can effectively reduce learning pressure:

*“It turns out that accounting classes can be so much fun”* (Interview Record Code: S-18-02)

*“It's very interesting, I can also fight monsters when I study accounting, I'm interested”* (Interview Record Code: S-24-03)

*“Since accounting combined with mobile games, I really feel that accounting is no longer a boring course!"* (Interview No.：S-08-04)

*“Playing games made me feel livelier in the course and made me more aware that accounting study can be better”* (Interview Record Code: S-36-02)

*“I feel that this game made by the teacher is very thoughtful and specially designed.”* (Interview Record Code: S-06-02)

*“Students will communicate with each other on how to fight monsters, and they will also take the initiative to ask me about gameplay and answers to questions, increasing the interaction between teachers and students.”* (Teaching Note: T-0530)

#### 6.3.3 The content of the game can strengthen the learning effect

Students agree that this teaching mode can effectively strengthen the memory and effect of the accounting subject content:

*“I think this mobile game made me learn a lot about accounting”* (Interview Record Code: S-9-02)

*“This mobile game makes me feel that learning accounting is much easier”* (Interview Record Code: S-14-01)

*“The game is very interesting and makes my study more efficient”* (Interview Record Code: S-07-03)

#### 6.3.4 Games play a role in promoting active learning

Students respond that they are willing to spend more time in active learning:

*“You can play it whenever you have time.”* (Interview Record Code: S-11-02)

“After the game is broken, borrow family members to play again without a mobile phone” (Interview Record Code: S-05-03)

*“Some students install the game on different mobile phones and increase their own practice, hoping to break the level perfectly, which makes me very happy and reflects the learning effect of the game.”* (Teaching Note: T-0605)

#### 6.3.5 Suggestions for the future improvement of accounting mobile games

*“I don't know how to use a mobile phone, the characters are a little small, and it's tiring to watch for too long”* (Interview Record Code: S-42-01)

*“There are many games in the accounting mobile game. It would be better if you could add more annotations. Otherwise, you must explain it again through the teacher.”* (Interview Record Code: S-02-03)

The feedback from the students on the quality of teaching shows that the students feel the benefits of game-based mobile learning that are not limited by time and space. Especially the students in the division of continuing education. In learning, reduce the pressure of learning. Through the repeated practice of games, let professional knowledge no longer be a book from heaven. Integrate games into the curriculum to improve learning interest and motivation. It encourages students to spend extra time on learning and stimulate students’ willingness to learn actively.

According to the content of students' study sheets, it is found that students' learning effectiveness has been significantly improved. At the same time, the reflection of teachers' teaching logs and classroom observations were collected as well. It is found that the introduction of game-based mobile learning in accounting classrooms can stimulate students' learning motivation. There are more opportunities for classroom interaction and practical application which not only makes learning more interesting but also significantly improves learning efficiency.

Thus, due to individual differences among classmates, some classmates should pay special attention to the areas where they need assistance when they have difficulty using the mobile game. Meanwhile, they are limited by the screen of the mobile phone. Moreover, the game description is expressed in an essential way and will be discussed and modified according to the suggestions of the students in the future to optimize the students' experience.

### 6.4 Discussion

Previous studies [15,21,70] mentioned that students thought that learning accounting is boring and difficult to understand, with mostly negative evaluations. Based on researchers' teaching experience and previous studies [35,71], students feared accounting learning and being used to absent courses as well. Regardless of whether they have learned accounting before. They thought that accounting knowledge was difficult for them. Most of them wished the accounting course could be designed easier to understand. During interaction with students, the researchers found that most have studied accounting before and have a mentality of exclusion towards accounting. They felt that there were lots of mathematical theories in accounting courses, and they also encountered bottlenecks in the calculation of given exercises. They never understand debit and credit in accounting, so they gave up easily after learning [72]. As a teacher, the researchers can also feel the pressure and pain of students deeply when their academic achievement in accounting is compared too low. To break through traditional teaching methods, this study provides a lively, interactive, and motivating teaching method for students. The self-developed accounting mobile game is integrated into accounting teaching so that students can learn accounting knowledge on mobile devices. The mobile device itself has mobility and is not restricted by geographical areas. It allows learners to study at anytime and anywhere. Thus, a ubiquitous learning environment is created. At the same time, the game is richer in the gamification mechanism and designing tasks based on the accounting theme. It is expected that the game can stimulate students' enthusiasm for learning and guide students to study independently. The prior studies [73,74] pointed out that students’ learning engagement is positively related to their learning effectiveness. Thus, students will improve their learning performance once they are willing to spend time in study. To solve the problems in the teaching field and make up for the gap in accounting research.

The results both gained from quantitative and qualitative data analysis significantly supported that the designed gamified mobile learning model will positively impact students' learning outcomes in accounting education. This result complied with related studies [52,53,[55], [56], [57]]. The only rejected prediction was the path of system quality to users' intention, this results against Alkhawaja et al.‘s [54] finding. Further, the partial mediation between user satisfaction, users' intention, and users' learning engagement are to be supported by analysis results in this study. The reason to be discussed by researchers is based on quantitative and qualitative data review and researchers' teaching note review.

Based on the results, the system quality could not affect students' intention to use appeared in Taiwan studies [75,76]. The curriculum requirements may cause the reason for this result that students were not aware of the designed game quality and the game didn't persecute them when they are using the game to learn accounting subject. Many studies argued that mobile game-based learning was a useful learning approach to enhance the efficiency of students' learning performance [5,26,27,36,57,62]. This perspective is supported by this study. Even though the factor ‘system quality’ without the prediction power affects students' intention to use the designed accounting game, however, this finding should be checked by future studies.

Further, user satisfaction plays a partial mediation effect between the intention to use and learning engagement significantly. Thus, using a mobile game-based learning method could inspire students to engage in accounting learning through the satisfaction of using a designed accounting game directly. The results of this study present a practical model for facilitating undergraduates' accounting learning with technological tools [26]. In general, the students responded positively to the developed teaching and learning model. Thus, the verified mobile game-based learning model is a useful solution to coping with students’ low motivation for accounting learning in Taiwan.

## 7 Conclusion and recommendation

This research developed an accounting mobile game, combined with the mobile teaching platform, to create a game-based mobile learning environment. An innovative course plan was constructed for accounting teaching and the course content was designed through an effective teaching model (BOPPPS) to assist students to understand accounting knowledge easily. Meanwhile, various classroom interactions were applied to enhance the learning effect. Quantitative and qualitative data collected in the course are analyzed by several analytical techniques. The curriculum improvement was implemented by teaching reflection to review and making rolling corrections to the problems. To the best of our knowledge, this is one of few studies of game-based mobile learning in accounting education to fill the gap in accounting education research.

The results of the study found that after the experience of game-based mobile learning, students' learning effect was significantly improved. Secondly, through the collection of game-based mobile learning experience data through questionnaires, most of the students have positive feedback on the experience of using accounting mobile games and further explore the impact of game-based mobile learning on accounting education through the successful model of information systems. The empirical results show that information quality and service quality have a significant positive impact on intention to use, and there is a significant positive correlation between intention to use, user satisfaction, and learning engagement. Intention to use has a direct positive impact on learning engagement, and user satisfaction also has a partial mediating effect on learning engagement. The results indicate that the accounting mobile game developed by this research integrated accounting knowledge into the game to provide students with useful, reliable, and convenient accounting learning information. When students used accounting mobile games to learn, the system would give feedback and rewards in a timely manner. If students make mistakes in the challenge, the system will also give correct answers, providing timeliness, responsiveness, and assurance. These two features strengthen students’ intention to use, thereby enhancing user satisfaction and learning engagement.

The research subjects of this study are the students from the division of continuing education usually at work, with limited study time, and from different fields and backgrounds. The accounting courses are even more unfamiliar to them, and the educational games are different from ordinary games. Students must be able to understand professional accounting knowledge in advance, then they can play the game successfully. The guidance of the teacher and the cooperation of the students are particularly important. Therefore, during the course, adjustments and corrections will be made according to the situation of the students. Using modern technology and innovative teaching methods requires patience to explain and communicate with the students so that they can learn online and enjoy the fun of learning. Meanwhile, teachers should care for, counsel, and arrange teaching assistants to aid students with learning problems. The design of teaching activities should not only consider the load level of students, but also be able to maintain students' enthusiasm for learning, guide students to acquire knowledge, and cultivate professional knowledge. Students are the protagonists of the classroom. Diversified course activities require students to have more learning engagement. The cooperation of the classmates is a key factor affecting the teaching effect. Due to the diverse composition of classmates, the course needs to be adjusted according to the situation of individual students. In addition, the support of hardware and software equipment is also important. Students sometimes reported that the game was stuck, the teaching platform could not be used, or the mobile phone capacity was limited, and there was no space to install the APP. It is necessary to eliminate the problem in a timely manner or provide other alternatives, such as bringing a tablet for students to use, etc. The status of software and hardware equipment would affect the learning experience of students.

The practical experience gained from the implementation of the course can provide teachers with a reference when implementing game-based mobile learning in the future, which can be helpful to accounting education. The subjects taught in this experiment are primary accounting, which cannot be deduced from other subjects such as intermediate accounting, management accounting, and auditing. In addition, the sampling of this study only deliberately selects specific students as the research objects, and there are limitations in the inference and validity of the research results. It is suggested that follow-up research can be extended to students from different universities or related institutions to make the research results more appropriate inference.

## Funding statement

This study was funded by Teaching Practice Research Program, 10.13039/100010002Ministry of Education, Taiwan (No. PBM1080125).

## Figures and Tables

**Table:** 

Structure	Items	Factor loading	t-value	Cronbach's α	rho_A	CR	AVE
Information quality	4	0.903–0.953	17..802–46.013	0.948	0.950	0.962	0.865
System quality	3	0.876–0.914	18.914–25.673	0.879	0.889	0.925	0.805
Service quality	3	0.912–0.977	17.231–104.025	0.947	0.948	0.966	0.904
Intention to use	3	0.922–0.958	23.475–53.742	0.929	0.932	0.955	0.876
User satisfaction	4	0.882–0.949	12.65–38.217	0.942	0.948	0.959	0.853
Learning input	4	0.888–0.953	17.802–68.536	0.940	0.942	0.957	0.848
