# Supplementary material for: The study on designed gamified mobile learning model to assess students’ learning outcome of accounting education
Source: Heliyon. 2023 Feb 10;9(2):e13409. doi: 10.1016/j.heliyon.2023.e13409 (PMC9958451; doi:10.1016/j.heliyon.2023.e13409)
Supplement: Multimedia component 1 [file mmc1.docx]

**References**

[1] Bado, N. (2022). Game-based learning pedagogy: A review of the literature. *Interactive Learning Environments, 30*(5), 936-948.

[2] Taiwan Communication Survey (2019). *Survey on Mobile Phone Usage Habits of Taiwanese-Ministry*. Issue 90. July 30, 2022. Retrieved from http://www.crctaiwan.nctu.edu.tw/epaper/%E7%AC%AC90%E6%9C%9F20190815.htm.

[3] Cardinot, A., & Fairfield, J. A. (2022). Game-based learning to engage students with physics and astronomy using a board game. In *Research Anthology on Developments in Gamification and Game-Based Learning* (pp. 785-801). IGI Global.

[4] Mao, W., Cui, Y., Chiu, M. M., & Lei, H. (2022). Effects of game-based learning on students' critical thinking: A meta-analysis. *Journal of Educational Computing Research, 59*(8), 1682-1708.

[5] Lei, H., Chiu, M. M., Wang, D., Wang, C., & Xie, T. (2022). Effects of game-based learning on students' achievement in science: A meta-analysis. *Journal of Educational Computing Research*, *60*(6), 1-17.

[6] Zhang, Q., & Yu, Z. (2022). Meta-Analysis on Investigating and Comparing the Effects on Learning Achievement and Motivation for Gamification and Game-Based Learning. *Education Research International*, *2022*, 1-19.

[7] Yu, J., Denham, A. R., & Searight, E. (2022). A systematic review of augmented reality game-based Learning in STEM education. *Educational Technology Research and Development*, *70*, 1169-1194.

[8] Wu, M. L. (2011). Meeting new challenges with digital action learning. *T&D, 124*, 1-20.

[9] Warman, P. (2018). *Newzoo cuts global games forecast for 2018 to $134.9 billion; lower mobile growth partially offset by very strong growth in console segment*. Retrieved on 11 April 2022 from https://newzoo.com/insights/articles/newzoo-cuts-global-games-forecast-for-2018-to-134-9-billion/.

[10] Zhan, Z. Z., Tong, Y., Lan, X., & Zhong, B. (2022). A systematic literature review of game-based learning in Artificial Intelligence education. *Interactive Learning Environments*, 1-22.

[11] Kantar Insights Taiwan (2022). *Male and female preferences are very different! How can Taiwan's mobile game market continue to grow*. Taipei, Insights Division, Kantar Taiwan. Retrieved July 30, 2022. Retrieved, from https://kantar.com.tw/News_detail.php?nid=44

[12] Seow, P. S., & Wong, S. P. (2016). Using a mobile gaming app to enhance accounting education. *Journal of Education for Business*, *91*(8), 434-439.

[13] Akkerman, S., Admiraal, W., & Huizenga, J. (2009). Storification in history education: A mobile game in and about medieval Amsterdam. *Computers & Education, 52*(2), 449-459.

[14] Carenys, J. & Moya, S. (2016). Digital game-based learning in accounting and business education: A literature review. *Accounting Education*, *25*(6), 598-651.

[15] Jamaluddin, J., Mahali, M., Din, N. M., Ahmad, M. A. N., Fadzillah, N. S. M., & Jabar, F. A. (2020). Students’ motivation level in gamification of accounting teaching and Learning – A case of ‘Accounting on the Block’. *Social and Management Research Journal, 17*(1), 17-34.

[16] Mousa, R. (2019). Addressing the AICPA core competencies through the usage of the monopoly™ board game. *Accounting Research Journal, 32*(2), 166-180.

[17] Schwabe, G., & Göth C. (2005). Mobile learning with a mobile game: Design and motivational Effects. *Journal of Computer Assisted Learning*, *21*(3), 204-216.

[18] da Silva, M. M., Teixeira, J. M. X., Cavalcante, P. S., & Teichrieb, V. (2019). Perspectives on how to evaluate augmented reality technology tools for education: A systematic review. *Journal of the Brazilian Computer Society,* *25*(1), 1-18.

[19] Bhavani, G., Mehta, A., & Dubey, S. (2020). Literature Review: Game Based Pedagogy in Accounting Education. *International Journal of Financial Research, 11*(6), 165-176.

[20] Nitkin, M. R. (2011). Game of business: A game for use in introductory accounting. *The Accounting Educators’ Journal*, *21*, 131–152.

[21] Calabor, M. S., Mora, A., & Moya, S. (2019). The future of 'serious games' in accounting education: A Delphi study. *Journal of Accounting Education, 46*, 43-52.

[22] Rebele, J. E., & Pierre, E. K. S. (2015). Stagnation in accounting education research. *Journal of Accounting Education*, *33*(2), 128-137.

[23] Ekmekci, O. (2013). Being there: Establishing instructor presence in an online learning environment. *Higher Education Studies, 3*(1), 29-38.

[24] Pattison, P., & Day, R. W. C. Eds. (2006). *Instructional Skills Workshop Handbook for Participants*. The Instructional Skills Workshop International Advisory Committee, TAG, UBC: Vancouver.

[25] Hou, H. Z. (2016). *Game-Based Learning: Start Self-Learning X Joyful Collaboration, Play Learning Together.* Taiwan: Family Lifestyle.

[26] Tavares, N. (2022). The use and impact of game-based learning on the learning experience and knowledge retention of nursing undergraduate students: A systematic literature review. *Nurse Education Today*, *117*, 1-6.

[27] Ahmed, A. A. A., Ampry, E. S., Komariah, A., Hassan, I., Thahir, I., Hussein Ali, M., ... & Zafarani, P. (2022). Investigating the effect of using game-based learning on EFL learners' motivation and anxiety. *Education Research International, 2022*, 1-9.

[28] Garris, R., Ahlers, R., & Driskell, J. E. (2002). Games, motivation, and learning: A research and practice model. *Simulation & Gaming*, *33*(4), 441-467.

[29] Bainbridge, K., Shute, V., Rahimi, S., Liu, Z., Slater, S., Baker, R. S., & D'Mello, S. K. (2022). Does embedding learning supports enhance transfer during game-based learning? *Learning and Instruction, 77*, 101547.

[30] Xu, D., & Wang, H. (2006). Intelligent agent supported personalization for virtual learning environments. *Decision Support Systems, 42*(2), 825-843.

[31] Ou, K. L., Zen, Y. T., & Yao, Y. J. (2014). Research on the influence of somatosensory digital game action learning system on learning achievement and learning retention. *Chinese Journal of Science Education*, *22*(2), 163-184.

[32] Walton, G., Childs, S., & Blenkinsopp, E. (2005). Using mobile technologies to give health students access to learning resources in the UK community setting. *Health Information & Libraries Journal, 22*, 51-65.

[33] Wang, P. (2013). Analysis of the support function and design principles of WeChat mobile learning. *Journal of Distance Education*, *6*, 34-41.

[34] Huang, W., Liu, J., Wang, X., Li, J., Zhang, R., & Liu, Y. (2016). Application of mobile learning and big data on improving flipped classroom and MOOCs. *Transactions on Computer Science and Engineering, 3*, 1-7.

[35] Sari, E. N., & Zamroni, Z. (2019). The impact of independent learning on students’ accounting learning outcomes at vocational high school. *Journal Pendidikan Vokasi, 9*(2), 141-150.

[36] Pan, Y. L. (2017). Discussion on the application of smart phone-based mobile learning in physical education teaching. *Taiwan Educational Review Monthly*, *6*(8), 177-206.

[37] Yan, C. H. (2015). *E-Learning/Concepts, Methods, Practice, Design, Implementation.* Taipei: Go top Information Inc.

[38] Hsiao, C. C., & Tu, C. H. (2012). The Developmental of "The Inventory of Teachers' Teaching for Creativity". *Curriculum and Teaching, 15*(2), 87 – 117.

[39] Segatto, W., Herzer, E., Mazzotti, C. L., Bittencourt, J. R., & Barbosa, J. (2008). Mobio threat: A mobile game based on the integration of wireless technologies. *Computers in Entertainment*, *6*(3), 1-14.

[40] Amory, A. (2001). Building an educational adventure game: Theory, design, and lessons. *Journal of Interactive Learning Research, 12*(2), 249-263.

[41] Rollings, A., & Morris, D. (2003). *Game architecture and design: A new edition. Indianapolis*. IN: New Riders

[42] Liu, T. Y., & Chu, Y. L. (2010). Using ubiquitous games in an English listening and speaking course: Impact on learning outcomes and motivation. *Computers & Education*, *55*(2), 630-643.

[43] Tsai, P. S. (2011). Japanese enterprise mobile learning application case - McDonald's uses NDS for employee training. *Digital Archives and Learning Newsletter*, *10*(12), 31-40.

[44] DeLone, W. H., & McLean, E. R. (1992). Information systems success: The quest for the dependent variable. *Information Systems Research*, *3*(1), 60-95.

[45] Mcgill, T., Hobbs, V., & Klobas, J. (2003). User-developed applications and information systems success: A test of DeLone and McLean’s model. *Information Resources Management Journal, 16*(1), 24–45.

[46] Seddon, P. B., Staples, S., Patnayakuni, R., & Bowtell, M. (1999). Dimensions of information systems success. *Communications of the Association for Information Systems, 2*(1), 20.

[47] Hamid, S., Waycott, J., Kurnia, S., & Chang, S. (2015). Understanding students' perceptions of the benefits of online social networking use for teaching and learning. *The Internet and higher education, 26*, 1-9.

[48] Webster, J., & Ahuja, J. S. (2006). Enhancing the Design of Web Navigation Systems: The Influence of User Disorientation on Engagement and Performance. *MIS Quarterly*, *30*(3), 661-678.

[49] Lalmas, M., O’Brien, H., & Yom-Tov, E. (2014). Measuring user engagement Synth. Lect. *Information Concepts, Retrieval, and Services*, *6*(4), 1-132

[50] DeLone, W. H., & McLean, E. R. (2003). The DeLone and McLean Model of Information Systems Success: A Ten-Year Update. *Journal of Management Information Systems*, *19*(4), 9-30.

[51] Reeve, J., & Tseng, C. M. (2011). Cortisol reactivity to a teacher’s motivating style: The biology of being controlled versus supporting autonomy *Motivation and Emotion, 35*, 63-74

[52] Lee, C. J., & Kim, C. (2014). An implementation study of a TPACK-based instructional design model in a technology integration course. *Educational Technology Research and Development, 62*(4), 437-460.

[53] Gharaibeh, M. K., & Gharaibeh, N. K. (2020). An empirical study on factors influencing the intention to use mobile learning. *Advances in Science, Technology and Engineering Systems Journal*, *5*(5), 1261-1265.

[54] Alkhawaja, M. I., Abd Halim, M. S., Abumandil, M. S., & Al-Adwan, A. S. J. C. E. T. (2022). System Quality and Student’s Acceptance of the E-learning System: The Serial Mediation of Perceived Usefulness and Intention to Use. *Contemporary Educational Technology,* *14*(2), 1-15.

[55] Mugion, R. G., Toni, M., Raharjo, H., Di Pietro, L., & Sebathu, S. P. (2018). Does the service quality of urban public transport enhance sustainable mobility? *Journal of Cleaner Production*, *174*, 1566-1587.

[56] Wan, L., Xie, S., & Shu, A. (2020). Toward an understanding of university students’ continued intention to use MOOCs: When UTAUT model meets TTF model. *Sage Open*, *10*(3), 1-15.

[57] Bitrián, P., Buil, I., & Catalán, S. (2021). Enhancing user engagement: The role of gamification in mobile apps. *Journal of Business Research*, *132*, 170-185.

[58] Coates, H. (2010). Development of the Australasian survey of student engagement (AUSSE). *Higher Education, 60*(1), 1-17.

[59] Blasco-Arcas, L., Buil, I., Hernández-Ortega, B., & Sese, F. J. (2013). Using clickers in class. The role of interactivity, active collaborative learning and engagement in learning performance. *Computers & Education, 62*, 102-110.

[60] Kuh, G. D. (2003). What we’re learning about student engagement from NSSE: Benchmarks for effective educational practices. Change: *The Magazine of Higher Learning*, *35*, 24-32

[61] Skinner, E., Furrer, C., Marchand, G., & Kindermann, T. (2008). Engagement and disaffection in the classroom: Part of a larger motivational dynamic? *Journal of Educational Psychology*, *100*(4), 765–781.

[62] Rice, J. W. (2012). The gamification of learning and instruction: Game-based methods and strategies for training and education. *International Journal of Gaming and Computer-Mediated Simulations, 4*(4), 81-83.

[63] Barata, G., Gama, S., Jorge, J., & Gonçalves, D. (2017). Studying student differentiation in gamified education: A long-term study. *Computers in Human Behavior, 71*, 550–585.

[64] Putz, L. M., Hofbauer, F., & Treiblmaier, H. (2020). Can gamification help to improve education? Findings from a longitudinal study. *Computers in Human Behavior, 110*, 1-12.

[65] Sarstedt, M., & Ringle, C. M. (2010). Treating unobserved heterogeneity in PLS path modeling: a comparison of FIMIX-PLS with different data analysis strategies. *Journal of Applied Statistics, 37*(8), 1299-1318.

[66] Pirouz, D. M. (2006). *An overview of partial least squares*. Retrieved July 8th, 2022, from https://papers.ssrn.com/sol3/papers.cfm?abstract_id=1631359

[67] Bentler, P. M., & Wu, E. J., C. (1993). *EQS/Windows user’s guide*. Los Angeles: BMDP Statistical Software.

[68] Fornell, C., & Larcker, D. F. (1981). Evaluating structural equation models with unobservable variables and measurement error. *Journal of Marketing Research, 18*(1), 39–50.

[69] Sobel, M. E. (1982). Asymptotic confidence intervals for indirect effects in structural equation models. *Sociological Methodology, 13*, 290-312.

[70] Moncada, S. M., & Moncada, T. P. (2014). Gamification of learning in accounting education. *Journal of Higher Education Theory and Practice, 14*(3), 1-9.

[71] Lucas, U., & Meyer, J. H. (2005). Towards a mapping of the student world: The identification of variation in students' conceptions of, and motivations to learn, introductory accounting. *The British Accounting Review, 37*(2), 177-204.

[72] Jaijairam, P. (2012). Engaging accounting students: How to teach principles of accounting in creative and exciting ways. *American Journal of Business Education, 5*(1), 75-78.

[73] Carini, R. M., Kuh, J. D., & Klein, S. P. (2006). Student engagement and student learning: Testing the linkages. *Research in Higher Education*, *47*, 1-32.

[74] Huang, C., & Yang, Y. (2021). Research on the Relationships Among Learning Motivation, Learning Engagement, and Learning Effectiveness. *The Educational Review*, *5*(6), 182-190.

[75] Fang, C. Y., & Chen, S. F. (2018). A study of using information system success model to explore computerized adaptive Chinese Characters Test System. *National Chiayi University Journal of the Educational Research, 41*, 25-72.

[76] Tsay, Y. J., Wu, S., & Chiang, B. C. (2001). The Research on the Model of Information Systems Success for the Hospitals. *Journal of Management and Business Research, 18*(2), 289-309.

**Appendix 1.** Summary table of individual reliability, component reliability, and mean extraction of variance coefficients (n=41)

| **Structure** | **Items** | **Factor loading** | **t-value** | **Cronbach’s α** | **rho_A** | **CR** | **AVE** |
| --- | --- | --- | --- | --- | --- | --- | --- |
| Information quality | 4 | 0.903~0.953 | 17..802~46.013 | 0.948 | 0.950 | 0.962 | 0.865 |
| System quality | 3 | 0.876~0.914 | 18.914~25.673 | 0.879 | 0.889 | 0.925 | 0.805 |
| Service quality | 3 | 0.912~0.977 | 17.231~104.025 | 0.947 | 0.948 | 0.966 | 0.904 |
| Intention to use | 3 | 0.922~0.958 | 23.475~53.742 | 0.929 | 0.932 | 0.955 | 0.876 |
| User satisfaction | 4 | 0.882~0.949 | 12.65~38.217 | 0.942 | 0.948 | 0.959 | 0.853 |
| Learning input | 4 | 0.888~0.953 | 17.802~68.536 | 0.940 | 0.942 | 0.957 | 0.848 |

**Appendix 2.** **Declaration of interest**

The authors have declared that no competing interests exist.

**Appendix 3.** **Data availability statement**

The original contributions presented in the study are included in the article/supplementary material, further inquiries can be directed to the corresponding authors.
